# The Profession of Nursing

**Published:** 1873

**Authors:** 


THE PROFESSION OF NURSING.—Every physician asserts that in critical cases good nursing is half the battle. A patient is down with typhoid, for instance. Day after day his pulse keeps up the same fierce, quick beat, the fever fiend pessesses his brain, life and strength seem to be ebbing away. The doctor comes in many times in twenty-four hours, watching, waiting, wary, pitting against the cruel disease every resource of his art; for the typhoid is like an Indian fighter in its treacheries and surprises. But the doctor has other patients and cannot stay in one sick-room all the time. He can give his directions minutely, prepare stimulants wisely, and order aconite and belladonna, acetate of lead or any of the remedies in the catalogue thoughtfully, but he must leave the carrying out of his instructions to other people. Those other people may be efficient, or they may not. It is frequently about as uncertain as the toss of a copper which they are. They may be sincerely desirous to do their best, and yet for want of knowledge or self-reliance, or by reason of their great anxiety for the patient's recovery, they may do their worst. Often when a physician leaves a person "doing well in the morning," he returns at night to find a retrogression, which he cannot say to the weary wife or mother, "You have brought about," yet which he plainly sees is due to unskilful nursing.—*Sanitarian..*

